# Optimized Jasmonic Acid Production by *Lasiodiplodia theobromae* Reveals Formation of Valuable Plant Secondary Metabolites

**DOI:** 10.1371/journal.pone.0167627

**Published:** 2016-12-01

**Authors:** Felipe Eng, Sven Haroth, Kirstin Feussner, Dorothea Meldau, Dmitrij Rekhter, Till Ischebeck, Florian Brodhun, Ivo Feussner

**Affiliations:** 1 Cuban Research Institute on Sugar Cane Byproducts, Vía Blanca & Carretera Central 804, San Miguel del Padrón, Havana, Cuba; 2 Georg-August-University Göttingen, Albrecht-von-Haller-Institute for Plant Sciences, Department of Plant Biochemistry, Göttingen, Germany; 3 Georg-August-University Göttingen, Göttingen Center for Molecular Biosciences (GZMB), Department of Plant Biochemistry, Göttingen, Germany; Universita degli Studi di Pisa, ITALY

## Abstract

Jasmonic acid is a plant hormone that can be produced by the fungus *Lasiodiplodia theobromae* via submerged fermentation. From a biotechnological perspective jasmonic acid is a valuable feedstock as its derivatives serve as important ingredients in different cosmetic products and in the future it may be used for pharmaceutical applications. The objective of this work was to improve the production of jasmonic acid by *L*. *theobromae* strain 2334. We observed that jasmonic acid formation is dependent on the culture volume. Moreover, cultures grown in medium containing potassium nitrate as nitrogen source produced higher amounts of jasmonic acid than analogous cultures supplemented with ammonium nitrate. When cultivated under optimal conditions for jasmonic acid production, *L*. *theobromae* secreted several secondary metabolites known from plants into the medium. Among those we found 3-oxo-2-(pent-2-enyl)-cyclopentane-1-butanoic acid (OPC-4) and hydroxy-jasmonic acid derivatives, respectively, suggesting that fungal jasmonate metabolism may involve similar reaction steps as that of plants. To characterize fungal growth and jasmonic acid-formation, we established a mathematical model describing both processes. This model may form the basis of industrial upscaling attempts. Importantly, it showed that jasmonic acid-formation is not associated to fungal growth. Therefore, this finding suggests that jasmonic acid, despite its enormous amount being produced upon fungal development, serves merely as secondary metabolite.

## Introduction

Due to the limited amount of exhaustable resources and the constantly increasing prices thereof renewable resources are of rising interest [1]. Besides oils and lipids that serve as essential raw material for chemical industry [2], also compounds of the secondary metabolism of different species are becoming an important focus in industrial research. Those compounds are often used as medicinal drugs. In addition, many novel constitutents of cosmetics, pharmaceuticals and nutraceuticals are developed on the physico-chemical basis of the core structures of secondary metabolites [3].

Jasmonates consitute one group of metabolites that is of economical importance. They are α-linolenic acid-derived compounds that exhibit a cyclopentanone ring as structural core-element to which one aliphatic and one carboxylic side chain is attached. The major representatives are jasmonic acid (JA), its methyl ester (MeJA) as well its isoleucine conjugate (JA-Ile). Jasmonates are widely distributed in algae [4], higher plants [5] and microorganisms [6]. Over the past decades a large body of research has been spend on the analysis of JA-function and JA-metabolism in plants and many details are known today. In plants jasmonates play important roles as growth inhibitors, they stimulate plant senescence, and they are also involved in flower development. Furthermore, they function as regulators for plant immunity that induces the expression of defensive genes after pathogen attack or feeding insects [7]. JA biosynthesis is catalyzed in two spatially separated cell compartments–the plastid and the peroxisome. In the former one the biosynthetic pathway is initiated by the peroxidation of α-linolenic acid derived from a plastidial membrane by the action of a 13*S*-lipoxygenase (13*S*-LOX). In the following reactions the hydroperoxy fatty acid is converted by allene-oxide synthase (AOS) and allene-oxide cyclase (AOC) to 12-oxo-phytodienoic acid (OPDA). This compound represents the first cyclic intermediate in this pathway. After its transport to the peroxisome, the ring system is reduced and the octanoic side chain is further processed by three rounds of β-oxidation finally yielding JA [8]. Besides this well-studied biosynthesis in plants, neither the function nor the biosynthetic pathways leading to JA in microorganisms are known yet.

MeJA was the first jasmonate that has been identified. It was originally isolated as an odoriferous constituent of the essential oil of *Jasminun grandiflorum* [9]. This compound is of special interest for perfume and flavor industry as it is an important component for many fragrance mixtures found in cosmetic (*i*. *e*. soaps, shampoos) and home care products (*i*. *e*. household cleaners) [10]. In the future, MeJA might also become more important for pharmaceutical industry as recent studies suggested that MeJA not only exhibits antidepressant, anti-aggressive activities in mammals but also shows anti-inflammatory effects comparable to that of prostaglandins [11]. Furthermore, growth of some cancerous cells lines of rats and humans can be suppressed by jasmonates *in vitro* and *in vivo* [12, 13]. These findings underline the potential use of those compounds as medicinal drugs.

Due to the low concentration necessary for hormonal function *in vivo*, plants naturally synthesize JA only in very small amounts. This makes the isolation of this compound for industrial purposes difficult and expensive. However, recent studies demonstrated that some fungal species are also capable of producing JAs in amounts exceeding those formed by plants [14–17]. Thus, it can be suggested that exploitation of those systems might be suitable for the industrial production of jasmonates. Different jasmonates have been detected in *Lasiodiplodia theobromae* (synonym *Botryodiplodia theobromae*) [18–21], *Fusarium oxysporum* [22], *Aspergillus niger* [23] and *Gibberella fujikuroi* [24]. However, details about biosynthetic routes of fungal JA-biosynthesis are still scarce and it is still unclear whether enzymatic pathways leading to formation of JA are similar to those in plants. Using a reverse genetic approach a specific 13*S*-LOX has recently been identified in *F*. *oxysporum* that might initiate JA-biosynthesis by catalysing the initial oxygenation reaction similar to that known from plants as described above [25]. Potential enzymes acting downstream of 13*S*-LOX—as for instance AOS or AOC in plants—have not been identified, yet. Hoffmann and co-worker reported, however, on the identification of a novel 9*R*-dioxygenase-AOS fusion enzyme that oxidizes linoleic acid to its corresponding 9*S*-hydroperoxy derivative and further isomerizes this intermediate yielding 9*S*(10)-epoxy-10,12-octadecadienoic acid in *F*. *oxysporum* [26]. Interestingly, a similar enzymatic activity has also been found in *L*. *theobromae* suggesting related metabolic functions in both fungi [27].

The objective of the present study was on the one hand to optimize the cultivation conditions of *L*. *theobromae* in order to increase JA yield and to obtain a kinetic model for fungal growth in respect to JA production. For this purpose, we not only investigated the influence of the different cultivation parameters on JA formation but also quantified the effect on metabolites that might be formed upstream and downstream from JA. On the other hand, a further objective was to identify the main compounds secreted by *L*. *theobromae* and thereby to gain more information about fungal secondary metabolism.

## Materials and Methods

### Microorganism

*L*. *theobromae* strain 2334 from the lnstituto Nacional de Investigaciones Fundamentales de la Agricultura Tropical (Cuba) isolated from sunflower, was used. The strain was stored on potato dextrose agar (PDA) slants with glycerol (50%) at 4°C and in mycelial discs on PDA with glycerol (17%) at -80°C.

### Culture medium

Culture medium with the following composition was used (in g/L): glucose, 50; KNO_3_, 8.9; KH_2_PO_4_, 2.0; KCl, 0.3; MgSO_4_∙7H_2_0, 0.6; FeSO_4_∙7H_2_0, 0.6; ZnSO_4_∙7H_2_0, 0.03; MnSO_4_∙7H_2_0, 0.003; CuSO_4_∙7H_2_0, 0.003; Na_2_MoO_4_∙2H_2_0, 0.003; yeast extract, 1.0. Before autoclaving, initial pH was adjusted to 5.5–5.6 with NaOH (0.1 M). The study on the effect of NH_4_NO_3_ as nitrogen source was carried out using the same nitrogen concentration (*n*(N) = 0.088 M) in the culture media as with KNO_3_ (*n*(N) = 0.088 M).

### Culture techniques

A sample of the stock culture was transferred to PDA plates and incubated for 3 d at 30°C. Five, ten or fifteen disks of mycelium (7 mm diameter) were used for inoculation of 25 mL, 50 mL or 100 mL of culture medium in 100 mL, 250 mL or 500 mL Erlenmeyer flasks, respectively. The medium was supplemented with either NH_4_NO_3_ or KNO_3_ as nitrogen source. Cultures were grown for 12 d in surface cultures at 30°C.

### Analytical methods

Biomass concentration was determined by dry weight after broth filtration on cellulose membrane (Rotilabo®-round filters, Type 12A, retention range 16 μm, Carl Roth &Co., Karlsruhe, Germany) followed by drying at 60°C for 24 h. Glucose content was determined by hexokinase method as described [28]. JAs and phytohormone extraction was performed as described previously with some minor modifications [29]. Metabolites were extracted from the culture filtrate at 4°C under constant shaking (200 rpm, 60 min) in the dark using 0.75 mL MeOH and 2.5 mL Methyl-tert-butylether (MTBE). For quantification, deuterated standards were added (50 ng D5-JA, 150 ng D5-OPDA, 50 ng D6-salicylic acid (SA) and 100 ng D5-indole acetic acid (IAA)). Prior to centrifugation 0.6 mL H_2_O were added to enhance phase separation. The upper phase was collected; the lower phase was re-extracted with 0.7 mL MeOH/H_2_O (3:2.5, *v/v*) and 1.3 mL MTBE. The combined phases were evaporated under streaming nitrogen to dryness and re-suspended in a solution of acetonitrile/water/acetic acid (20/80/0.1, *v/v/v*). The analysis was performed using a 1100 HPLC system (Agilent Technologies, Santa Clara, USA) coupled to a 3200 hybrid triple quadrupole (ABSciex, Framingham, USA) as reported [30]. Mass transitions (in Da) were as follows: 214/62 for D5-JA, 209/59 for JA; 225/59 for 11/12-hydroxy-JA, 237/165 for OPC-4, 296/170 for D5-OPDA, 291/165 for cis-OPDA, 179/135 for D5-IAA, 174/130 for IAA, 160/116 for (indole carboxylic acid) ICA, 137/93 for SA, 141/97 for D6-SA and 207/137 for chorismate.

Structure confirmation of OPC-4 and 11/12-hydroxy-JA was well as structure elucidation of compounds 1–7 were done by high resolution MS/MS fragmentation studies with an ultra-high performance chromatography-electrospray ionization-quadrupole time-of-flight-mass spectrometer (UHPLC-ESI-QTOF-MS). A LC 1290 Infinity (Agilent Technologies, Karlsruhe, Germany) coupled with a 6540 UHD Accurate-Mass Q-TOF LC MS instrument with Jet Stream Technology as ESI source (Agilent Technologies, Karlsruhe, Germany) was used for all analyses. For LC an ACQUITY UPLC HSS T3 column (2.1 x 100 mm, 1.8 μm particle size, Waters Corporation, Milford, USA) was used at a flow rate of 0.5 mL/min at a temperature of 40°C. The solvent system consists of solvent A (water/formic acid (100/0.1, *v/v*) and solvent B (acetonitrile/formic acid (100/0.1, *v/v*). The following gradient was applied: 0–3 min from 1% to 20% solvent B, 3–8 min from 20% to 100% solvent B, 8–12 min 100% solvent B. The Q-TOF MS instrument was operated in Extended Dynamic Range and targeted MS/MS mode with a frequency of 2 GHz. Source conditions were: gas temperature: 250°C; drying gas flow: 8 L/min; nebulizer pressure: 35 psi; sheath gas temperature: 300°C; sheath gas flow: 8 L/min; VCap voltage: 3 kV; nozzle voltage: 200 V; fragmentor voltage: 100 V. Samples were ionized in negative and/or positive ESI mode with collision energy 10 eV. Data were acquired by Mass Hunter Workstation Acquisition software B.06 (Agilent Technologies, Karlsruhe, Germany) and analyzed by Mass Hunter Qualitative Analysis B.06 (Agilent Technologies, Karlsruhe, Germany).

## Results

### Identification of a potential JA precursor and oxidized JA derivatives

Prior to our optimization approach, we were interested in potential metabolites that might be formed up-stream and down-stream of JA in order to gain a deeper understanding of fungal JA-metabolism. Because in plants OPDA and specific 3-oxo-2-(pent-2-enyl)-cyclopentane-1-carboxylic acid (OPC)-derivatives are prominent JA-precursors, we primarily focused on the identification of those compounds. Moreover, we considered that oxidized JA-derivatives may be formed as catabolic JA-products similar to those that have been observed in plants [31–34].

**Fig 1:**
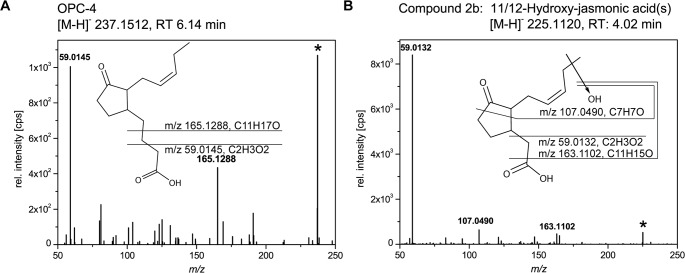
UHPLC-ESI-QTOF-MS analysis of OPC-4 and 11/12-hydroxy-JA formed by *L*. *theobromae* strain 2334. The fungus was cultivated in medium supplemented with KNO_3_ for 9 days (d). After extraction compounds of the culture filtrate were analyzed via UHPLC-ESI-QTOF-MS. Shown are high resolution MS/MS spectra (negative ionization mode; collision energy 10 eV) of (**A**) OPC-4 ([M-H]^-^ 237.1512, RT 6.14 min) and (**B**) 11/12-hydroxy-JA ([M-H]^-^ 225.1120, RT 4.02 min). Proposed fragmentation is shown as inset. Abbreviations: JA, jasmonic acid; OPC-4, 3-oxo-2-(pent-2-enyl)-cyclopentane-1-butanoic acid.

In search for those compounds we analysed the culture filtrate of *L*. *theobromae* strain 2334 by using UHPLC-ESI-QTOF-MS. We observed formation of 3-oxo-2-(pent-2-enyl)-cyclopentane-1-butanoic acid (OPC-4) and hydroxy-derivatives of JA (Fig 1), whereas OPDA could only be detected in trace amounts. The chemical structure of OPC-4 was confirmed by retention time and high resolution MS/MS-spectra as the corresponding authentic standard (Fig 1A). The parent ion represents the base peak at *m/z* 237.1512. Two diagnostic signals were at *m/z* 59.0145 (carboxy-methyl group) and *m/z* 165.1288, which corresponds to the sum formula C_11_H_17_O^-^ (Fig 1A) [35].

The MS/MS-spectrum of hydroxy-JA (compound 2b) is shown in Fig 1B. This compound exhibited an accurate mass of [M-H]^-^ 225.1120 and eluted at 4.02 min. The respective MS/MS-spectra displayed three fragment ions characteristic for JA-derivatives in the negative ionization mode. The base-peak was found at *m/z* 59.0132, which corresponds to the *m/z* of the carboxy-methyl group from the JA-backbone. It has been reported before that formation of this fragment is diagnostic for JA-derivatives [36]. An additional characteristic signal at *m/z* 163.1102 belongs to the fragment ion of [M-CO_2_-H_2_O-H]^-^, which results from neutral loss of the carboxyl-group as CO_2_ and the hydroxy-group as water. The signal at *m/z* 107.0490 arises from a cleavage of the cyclopentanone ring of the JA-backbone and the loss of hydroxy-group in C11 or C12 position yielding a C_7_H_7_O^—^fragment as indicated in Fig 1B. Based on this result, we assigned this metabolite as a hydroxy-JA. In order to evaluate the position of the hydroxy-group we used authentic 11-hydroxy- and 12-hydroxy-JA as standard. Both compounds eluted at the same retention time as compound 2b and gave the identical MS/MS-spectra suggesting that *L*. *theobromae* might either form 11-hydroxy, 12-hydroxy- or even both hydroxylated JA isomers. 11- and 12-hydroxy-JAs were described by Miersch and co-workers [19]. For this reason, we termed this peak 11/12-hydroxy-JA.

### Influence of cultivation conditions on fungal growth, production of JA, OPC-4, and 11/12-hydroxy-JA

**Fig 2:**
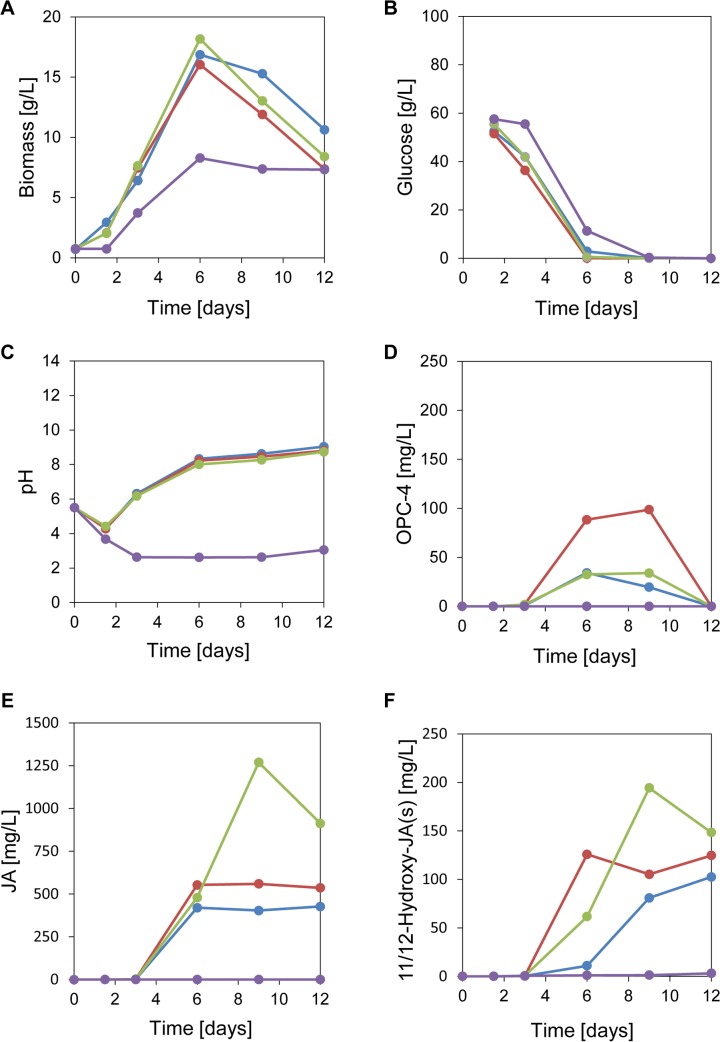
Time-course of biomass formation (**A**), glucose consumption (**B**), pH-changes (**C**), OPC-4-formation (**D**), JA-formation (**E**) and 11/12-hydroxy-JA formation (**F**) by *L*. *theobromae* strain 2334. Cultures were grown in 100 mL (blue line), 250 mL (red line) and 500 mL (green line) flasks with KNO_3_ as nitrogen source. Additionally, cultures were cultivated in 250 mL flasks containing medium supplemented with NH_4_NO_3_ as nitrogen source (violet line). Data are mean of 2–4 replicates. Two replicates: Red– 1.5 d, green– 1.5/3/6 d, violet 1.5 d and the glucose consumption. Four replicates: Red/green/violet– 12 d. The other data represent three replicates. Abbreviations: JA, jasmonic acid; OPC-4, 3-oxo-2-(pent-2-enyl)-cyclopentane-1-butanoic acid.

Under static culture conditions *L*. *theobromae* forms a mat on the surface of the culture medium [14]. Therefore, we started our optimization approach by investigating the influence of the culture volume and the associated surface area on biomass-production, glucose consumption, pH-changes and JA-metabolism. For this purpose, we cultivated *L*. *theobromae* strain 2334 in shake flasks of different sizes (100 mL, 250 mL and 500 mL) using culture medium supplemented with KNO_3_ as nitrogen source and monitored the different parameters mentioned above. In order to evaluate the influence of the nitrogen source on fungal growth and JA-metabolism, we performed additional experiments using culture medium that was supplemented with NH_4_NO_3_ instead of KNO_3._ The results are presented in Fig 2.

When KNO_3_ was used, the fungal cultures reached the stationary phase after 6 days (d) and decreased at the following time-points (Fig 2A). The biomass-yield, basing on glucose as substrate (Yx/s, g biomass/g glucose) gave similar results for the different culture volumes: 100 mL, Yx/s = 0.32; 250 mL, Yx/s = 0.31; 500 mL, Yx/s = 0.35. This suggests that fungal growth was independent from the culture volume. However, when NH_4_NO_3_ was used as nitrogen source the fungal biomass-yield was reduced by a factor of 2 (Yx/s = 0.15).

Temporal changes of the glucose uptake showed an inverse trend as the biomass accumulation (Fig 2B). When cultures were grown in the presence of KNO_3_ or NH_4_NO_3_, glucose was completely consumed within 6 d and 9 d, respectively. Within the same time window of day 1 to day 6, we observed drastic pH-changes that were dependent on the nitrogen source: whereas the pH immediately dropped to pH 3, when the fungus was grown in medium supplemented with NH_4_NO_3_, a pH increase to pH 8 was observed when KNO_3_ was supplied (Fig 2C).

Next, we were interested in the metabolic consequences of the different cultivation conditions with regard to JA, its potential precursors OPDA and OPC-4, as well as its potential catabolic derivative 11/12-hydroxy-JA. For this purpose, we analysed the culture filtrate at different time-points of cultivation [30]. We not only detected formation of OPC-4 and 11/12-hydroxy-JA but also found trace amounts of OPDA. However, this compound was measured in very low concentrations (< 0.01 mg/L) and could only be detected after 3 d of cultivation. At the same time point, OPC-4 started to accumulate (Fig 2D). From day 6 to day 8, OPC-4-formation was maximal and declined during the following time points (day 9—day 12) when KNO_3_ was used as nitrogen source. The amount of OPC-4 that was formed over the whole time course was similar for cultures grown in 100 mL and 500 mL flasks. However, when grown under identical conditions but in 250 mL flasks, the amount of OPC-4 was increased by a factor of 2–3. When NH_4_NO_3_ was supplied as a nitrogen source, no OPC-4 production could be observed. JA accumulation started in cultures supplemented with KNO_3_ approximately at day 3 (Fig 2E). Here, we interestingly observed that the culture size directly affected the amount of JA been synthesized. In cultures grown in 100 mL and 250 mL flasks, JA accumulated after 6–12 d to approx. 420 mg/L and 550 mg/L, respectively, which corresponds to a final product yield of Yp/s = 0.01 mg JA/g glucose. However, when grown in 500 mL flasks *L*. *theobromae* strain 2334 formed up to 1250 mg/L JA corresponding to a product yield of Yp/s = 0.03 mg JA/g glucose. For cultures grown in medium supplemented with NH_4_NO_3_ formation of JA was highly reduced, yielding only levels of 0.17 – 0.4 mg/L which corresponds to a product yield of Yp/s = 10 ng JA/g glucose. Beside the formation of JA and its potential precursors OPC-4 and OPDA, we also observed formation of the oxidized JA-derivative(s) 11/12-hydroxy-JA. In the plant field, a growing body of evidence suggests that this compound is part of the JA catabolic pathway [31, 33, 37].

Formation of 11/12-hydoxy-JA was also observed to start approx. at 3 d (Fig 2F). Here, we found that the culture volume directly influenced the maximal amount of 11/12-hydroxy-JA that had been formed. Up to 200 mg/L of 11/12-hydroxy-JA was formed by *L*. *theobromae* strain 2334 when cultivated in 500 mL flasks with KNO_3_ as nitrogen source. In smaller flasks (*i*.*e*. 100 mL and 250 mL) the amount of this metabolite was reduced by a factor of 1.5–2.

### Quantification of plant secondary metabolites produced by *L*. *theobromae* strain 2334 under different cultivation conditions

Next we measured plant hormones in extracts obtained from the culture filtrate. We detected very high amounts of indole-3-carboxylic acid (ICA) and small amounts of salicylic acid (SA) [30](Table 1). When KNO_3_ was used as nitrogen source, production of both compounds increased with the culture volume. Here, the concentration of ICA in the 500 mL culture after 9 days was with 2825 mg/L even higher than the respective amount of JA (Fig 2E). The amounts of SA were much lower but still increasing with the culture volume. In the presence of NH_4_NO_3_ the amounts of ICA and SA were strongly reduced. Conclusively, production of ICA as well as SA followed a similar trend as JA-production and increased when the culture size was increased.

**Table 1:** Average concentrations of plant secondary metabolites, indole-3-carboxylic acid (ICA) and salicylic acid (SA) in the culture medium of *L*. *theobromae* strain 2334. Culture filtrate was obtained after 9 days of growth in medium containing KNO_3_ as nitrogen source and cultivated as surface culture of different volumes: 100 mL, 250 mL and 500 mL. An additional experiment was performed as surface culture of 250 mL using medium with NH_4_NO_3_ as nitrogen source (250 ml-NH_4_NO_3_). Data are means ± standard deviations of three replicates. Detection limit for SA was 1x10^-4^ mg/L at a signal to noise ratio of three.

Yield (mg/L)				
Phytohormone	100 ml	250 ml	500 ml	250 ml-NH_4_NO_3_
ICA	269.25 ± 129.62	347.38 ± 78.20	2825.32 ± 276.11	0.19 ± 0.21
SA	0.81 ± 0.07	1.08 ± 0.30	4.67± 5.02	0.75 x 10^−3^± 5 x 10^−5^

### Modelling of cellular growth and JA production reveals JA as a fungal secondary metabolite

An attempt of modelling the cellular growth was also carried out by fitting the experimental data to a logistic model (Eq 1) where *X* is the biomass (g/L) at a specific moment of the cultivation time *t* (h), *Xo* and *Xmax* are the initial and maximum biomass concentration and μ_*m*_ the maximum specific growth rate (h^-1^). The experimental data for cultures grown in KNO_3_ containing medium described above were fitted with the logistic model through STATGRAPHIC Centurion version 15.1.0.2 (Stat Point Inc.) that uses the Marquardt-Levenberg algorithm to determine the parameters.

$$X=\frac{Xmax}{1+e^{\left(\ln\left(\frac{Xmax}{Xo}-1\right)-\mu mt\right)}}$$ (1)

The obtained values defining the model are presented in Table 2 for the different culture volumes. These data illustrate that fungal growth was not significantly affected by the different culture volumes. In a similar approach, we next modelled the time-dependent formation of JA using a logistic model (Eq 2), in which *P* is the JA production at a specific moment of cultivation time (*t*), *Po* and *Pmax* the initial and maximum JA production, and *μp* the maximum relative JA production rate.

$$P=\frac{Pmax}{1+e^{\left(\ln\left(\frac{Pmax}{Po}-1\right)-\mu pt\right)}}$$ (2)

**Table 2:** Parameters defining the logistic model of fungal growth. *L*. *theobromae* strain 2334 was cultivated in shake flasks of 100 mL, 250 mL and 500 mL in the presence of KNO_3_ as nitrogen source.

Growth Parameters	100 ml	250 ml	500 ml
Xo (g/L)	0.70	0.74	0.78
Xmax (g/L)	16.1	18.6	17.5
*μ*_*m*_ (h^-1^)	0.04	0.04	0.04
Regression Coefficient R^2^	0.972	0.982	0.981

The values of the equation parameters are shown in Table 3: While the maximal relative production rate *μp* observed for the different cultivation conditions was highly similar (0.07–0.06 h^-1^). The maximum of JA production increased with increased culture volume.

**Table 3:** Parameters defining the logistic model of JA-production. *L*. *theobromae* strain 2334 was cultivated in shake flasks of 100 mL, 250 mL and 500 mL in the presence of KNO_3_ as nitrogen source.

JA production parameters	100 ml	250 ml	500 ml
Po (mg/L)	0.11	0.15	0.11
Pmax (mg/L)	420	768	1357
*μ*_*P*_ (h^-1^)	0.07	0.06	0.06
Regression Coefficient R^2^	0.991	0.997	0.997

The correlation between growth and metabolite production can be portrayed by the Luedeking and Piret model [38] and it is represented by the following equation:

$$r_{P}=mX+{nr}_{X}$$ (3)

with *r*_*X*_ and *r*_*P*_ being the biomass and metabolite production rate, *X* the biomass concentration and *m* and *n* the parameters of the model. The model can also be expressed in an integrated form [39], as result of combining Eqs 1 and 3 as follows:

$$P=Po+mXo\left\{\frac{e^{\mu mt}}{\left[1,0-(\frac{Xo}{Xmax})(1,0-e^{\mu mt}\right]}-1\right\}+n\left(\frac{Xmax}{\mu m}\right)\;ln\;\left[1,0-(\frac{Xo}{Xmax})(1,0-e^{\mu mt})\right]$$ (4)

*Xmax*, *Xo*, *μm*, *P* and *Po* are the parameters defined in the logistic model, *m* and *n* the growth and non-growth associated parameters. According of the values of *m* and *n* the products of the process are classified as growth associated (*m* > 0 and *n* = 0), non-growth associated (*m* = 0, *n* > 0) or mixed growth associated (*m* > 0 and *n* > 0). The parameters *m* and *n* of the model were determined this way and are summarized in Table 4. The obtained data demonstrate that JA-production is a non-growth associated process. This finding thus clearly suggests that JA produced by *L*. *theobromae* strain 2334 is a typical fungal secondary metabolite.

**Table 4:** Parameters defining the Leudiking and Piret model in shake flasks of *L*. *theobromae* strain 2334 of volumes of 100 mL, 250 mL and 500 mL and in the presence of KNO_3_ as nitrogen source.

Parameters of logistic model	100 ml	250 ml	500 ml
Po (mg/L)	0	0	0
*m* (mg/g biomass)	0	0	0
*n* (mg/g biomass/h)	3.70	3.15	5.79
Regression Coefficient R^2^	0.966	0.989	0.980

The integrated form of the modified form of the Luedeking-Piret equation [40] was used to describe the substrate consumption over time (Eq 5).

$$So-S(t)=\alpha [X(t)-Xo]+\beta \left(\frac{Xmax}{\mu }\right)\;ln\;\left[1,0-(\frac{Xo}{Xmax})(1,0-e^{\mu t})\right]$$ (5)

where α and β, are the Luedeking-Piret equation parameters for substrate consumption.

As shown in Table 5 we found high regression coefficients R^2^ that indicate a good agreement of the experimental data obtained and the calculated functions. Thus, we are confident that the use of the logistic model to describe the fermentation-process of *L*. *theobromae* strain 2334 with regard to biomass-production, JA-formation, and substrate consumption was adequate.

**Table 5:** Substrate parameters defining of the Leudiking and Piret model in shake cultures of *L*. *theobromae* strain 2334 in volumes of 100, 250 and 500 mL.

Substrate parameters	100 ml	250 ml	500 ml
So (mg/L)	60.8	57.8	58.9
α (gS/L/g biomass)	3.80	2.94	2.20
β (gS/L/g biomass/h)	2.54 x 10^−4^	1.53 x 10^−3^	4.02 x 10^−3^
Regression Coefficient R^2^	0.980	0.999	0.999

### Identification of main secondary metabolites of *L*. *theobromae* strain 2334 culture filtrate

**Fig 3:**
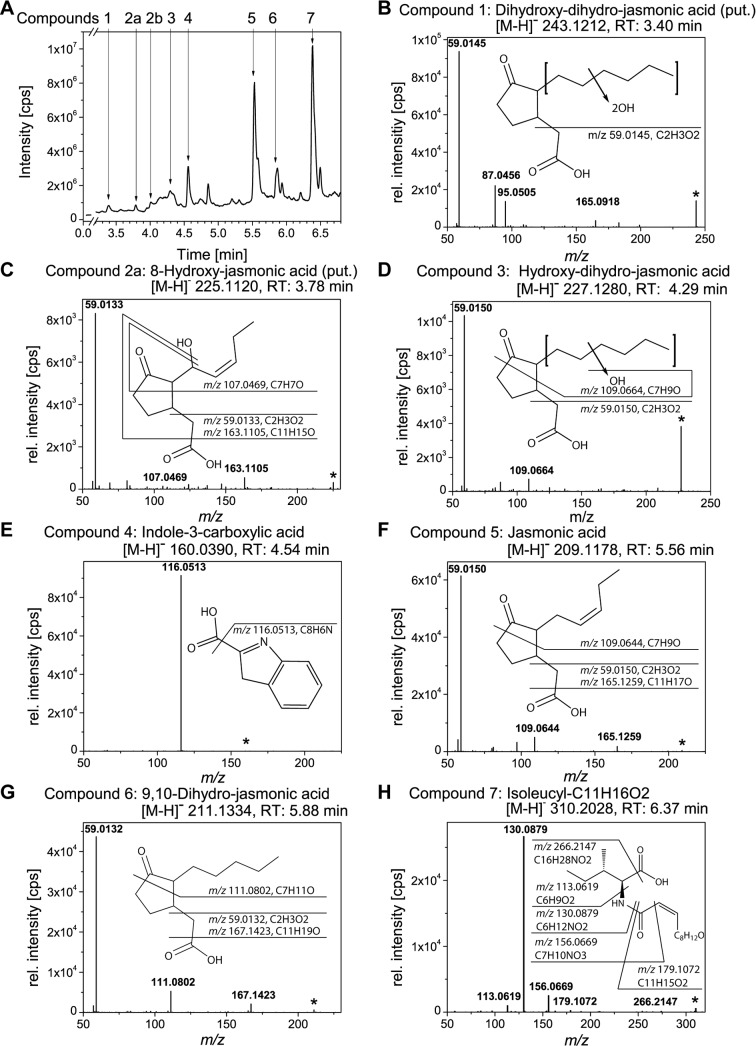
UHPLC-ESI-QTOF-MS analysis of the main metabolites in the culture medium of *L*. *theobromae* strain 2334. *L*. *theobromae* strain 2334 was cultivated in medium supplemented with KNO_3_ for 9 d. After extraction, compounds of the culture filtrate were analyzed via UHPLC-ESI-QTOF-MS. Shown is a total ion chromatogram (**A**), as well as high resolution MS/MS-spectra (negative ionization mode; collision energy 10 eV) of different compounds (**B-H**). Proposed structures and fragmentations are shown as inset for each spectrum. The following compounds were identified: dihydroxy-dihydro-JA ([M-H]^-^ 243.1212, RT 3.40 min) **(B**), 8-hydroxy-JA ([M-H]^-^ 225.1120, RT 3.78 min) (**C**), hydroxy-dihydro-JA ([M-H]^-^ 227.1280, RT 4.29 min) (**D**), Indol-3-carboxylic acid ([M-H]^-^ 160.0390, RT 4.54 min) **(E**), JA ([M-H]^-^ 209.1178, RT 5.56 min) (**F**), 9,10-dihydro-JA ([M-H]^-^ 211.1334, RT 5.88 min) (**G**), Isoleucyl-C_11_H_16_O_2_. ([M-H]^-^ 310.2028, RT 6.37 min) (**H**). Abbreviations: JA, jasmonic acid.

We were interested in the identification of further secondary metabolites that were produced by *L*. *theobromae* strain 2334 under conditions optimal for JA-biosynthesis. After extraction from the medium, we analyzed the different metabolites by using UHPLC-ESI-QTOF MS. A total ion chromatogram representative for the MS only mode of the UHPLC-ESI-QTOF-MS analysis is presented in Fig 3A. The majority of the compounds eluted from 3.4 min to 7.0 min. On the basis of the accurate mass and the MS/MS fragmentation patterns, we tentatively assigned the chemical structure of seven of those metabolites (compound 1–7, Fig 3). Compound 1 eluted at 3.40 min and exhibited an accurate mass signal of [M-H]^-^ 243.121 (C_12_H_19_O_5_^-^, Fig 3B). The MS/MS-spectrum of this compound showed the base-peak at *m/z* 59.0145. Representing the diagnostic fragment of the carboxy-methyl group from the JA-backbone, this suggests that compound 1 is a JA-derivative [36]. The signal at *m/z* 165.0918 (C_10_H_13_O_2_) is assumed to result from the loss the carboxy-methyl group as well as the loss of water from one of the proposed hydroxyl residues. In addition two fragment ions of *m/z* 87.0456 and *m/z* 95.0505 were detected, which represent fragments of the sum formulas of C_4_H_7_O_2_^-^ and C_6_H_7_O^-^, respectively. We tentatively assign this compound as a dihydroxy-9,10-dihydro-JA. However, the analysis did not allow to determine the exact position of both hydroxy-groups.

The following two compounds eluted at 3.78 min and at 4.02 min, respectively (Fig 3A, compound 2a and 2b). Both exhibited an identical accurate mass of [M-H]^-^ 225.1120 and gave similar MS/MS-spectra (compound 2a (Fig 3C) and 2b (Fig 1B)). Comparison of the retention times with that of authentic standards suggested that compound 2b is either 11- or 12-hydroxy-JA or a mixture of both (11/12-hydroxy-JA, see above). The chemical structure of compound 2a could not be fully resolved with respect to the position of the hydroxy-group. However, the shorter retention time suggests that the hydroxy group is located at the C8 as indicated in the inset of Fig 3C. 8-hydroxy-JA was described in the supernatant of *L*. *theobromae* by Miersch and co-workers [19].

The MS/MS-analysis of compound 3 is shown in Fig 3D. This compound eluted at 4.29 min with an accurate mass signal of [M-H]^-^ 227.1280. The MS/MS-spectrum resembled that of compound 2a and 2b. Again, we found the base-peak at *m/z* 59.0150 (carboxy-methyl group) suggesting that this compound is also a JA-derivative. The fragment ion at *m/z* 109.0664 (corresponding to the sum formula of C_7_H_9_O^-^) is shifted by the mass of two hydrogens to higher *m/z-*ratios compared to that of hydroxy-JAs (Fig 1B) and (Fig 3C). Those findings allowed the tentative assignment of compound 3 as a hydroxy-dihydro-JA.

Compound 4 eluted at 4.54 min and exhibited an accurate mass signal of [M-H]^-^ 160.0390. The MS/MS-spectrum (Fig 3E) displayed a highly intense base peak at *m/z* 116.0513 ([M-CO_2_-H]^-^, neutral loss of the carboxyl-group as CO_2_) and a very weak signal of the parent-ion at *m/z* 160.0390 [M-H]^-^. Comparison of the fragmentation pattern of compound 4 with that for ICA (Scripps Center for Metabolomics, METLIN data base: MID3795) confirmed the occurrence of ICA in the supernatant of *L*. *theobromae*.

Compound 5 (RT 5.56 min and [M-H]^-^ 209.1178) was identified as JA (Fig 3F). In accordance with the spectra of compound 1, 2, and 3, the base peak was again at *m/z* 59.0150 and additional signals were found at *m/z* 109.0644 and *m/z* 165.1259. The latter one had been described as JA-fragment of minor intensity (Scripps Center for Metabolomics, METLIN data base: MID 3345) [41]. In addition, the identity of JA was confirmed by comparing the retention time with that of an authentic standard.

MS/MS-spectrum of compound 6 (RT 5.88 min, [M-H]^-^ 211.1334, Fig 3G) was similar to that of JA in respect to the base-peak at *m/z* 59.0132. The minor signals of *m/z* 167.1423 and *m/z* 111.0802 were shifted to higher *m/z*-values by the mass of two hydrogens in comparison to the fragments of JA. Based on this result we assigned this compound as dihydro-JA.

Compound 7 eluted at 6.37 min and exhibited an accurate mass signal of [M-H]^-^ 310.2028 (Fig 3H). In order to elucidate the chemical structure of this compound, we performed MS/MS-experiments in the negative and positive ionization mode. However, both analyses together did allow the unequivocal identification of this metabolite. The MS/MS-spectrum of the negatively ionized analyte displayed a base peak at *m/z* 130.0879, which is characteristic for an isoleucine (Ile) or leucine (Leu) moiety. The signals at *m/z* 113.0619 (Ile/Leu moiety with a neutral loss of the amino group) as well as *m/z* 179.1072 ([M-Ile/Leu-H]^-^) confirmed the substructure. The fragment of *m/z* 156.0669 allowed to foretell the connection of a fatty acid like moiety (C_7_H_10_NO_3_^-^) via an amide (peptide) bound to the amino group of the Ile residue. By pseudo MS^3^ of *m/z* 132.1020 (Ile/Leu moiety) in the positive mode, Ile could be unequivocally identified as amino acid moiety of compound 7 (presence of the diagnostic Ile-fragment of *m/z* 41.0388, data not shown). In the positive MS/MS mode compound 7 showed a base-peak at *m/z* 266.2102 and additional signals at *m/z* 86.0966, *m/z* 125.2102 and *m/z* 198.1467 (data not shown). From all obtained data, we propose a chemical structure of this compound as shown in Fig 3H. The structure comprises of a C_11_H_15_O_2_ unit that is bound to the α-amino-group of an Ile-moiety via an amide bond. Due to an incomplete fragmentation of the proposed fatty acid like structure (C_11_H_15_O_2_ unit) by ESI-MS/MS we could not obtain further structural information.

## Discussion

In the present study, we aimed to improve the capacity of *L*. *theobromae* strain 2334 to produce JA, JA-derivatives and further secondary metabolites. Here, the focus laid primarily on the analysis of the influence of the nitrogen-source (KNO_3_ and NH_4_NO_3_) and the culture-volume. Only in medium containing KNO_3_, formation of JA was observed indicating that the choice of the nitrogen source is an important parameter for JA biosynthesis. Our results further demonstrate that JA production is highly dependent on the culture volume. We observed that the use of 500 mL flasks for cultivation led to the formation of up to 1.25 g/L JA after 9 days. This may be explained by an optimal oxygen and nutrient diffusion within the culture medium under these conditions. A similar trend has previously been reported by Dhandhukia and co-workers who obtained up to 170 mg/L JA when *L*. *theobromae* strain MTCC-3068 was grown in 1 L flasks [14]. This amount is reduced by a factor of 7–8 compared to JA formed by the *L*. *theobromae* strain used in this study. One explanation for this significant difference might be the varying inherent capacities of the two *L*. *theobromae* strains to synthesize JA. On the other hand, Dhandhukia and co-workers used NaNO_3_ as nitrogen source [14] whereas we used KNO_3_ for cultivation. As discussed above and also reported elsewhere the choice of nutritional parameters might directly influence the amount of JA produced by *L*. *theobromae* [15].

To deepen our understanding on JA-production by *L*. *theobromae* and thus to possibly further enhance the amount of JA that is formed, we additionally investigated the anabolism and catabolism of JA. For this purpose we analyzed the temporal changes in the amount of JA and also examined the cultures for metabolites that might be formed upstream (e.g. OPDA and OPC-4) or downstream of JA (hydroxy-JA derivatives) and monitored temporal alterations in their production. Interestingly, we detected the formation of OPDA at day 3 albeit in very minor concentrations (data not shown). Free OPDA has not been detected in *L*. *theobromae* before. In 2010, however, Tsukada and co-workers observed the transient production of the methyl-ester of OPDA [42]. From this finding it was hypothesized that *L*. *theobromae* might synthesize JA via a pathway analogous to that from plants [42]. In order to evaluate this idea, we further analyzed the cultivation medium and detected at defined time-points of growth significant amounts of OPC-4 (Fig 1A and Fig 2D). In plants, this metabolite presents the direct precursor of JA that is formed from OPDA after reduction and two-rounds of β-oxidation. Using the cultivation conditions described above, *L*. *theobromae* strain 2334 formed OPC-4 from day 3 to day 12 (Fig 2D). Within the same time-window, JA-accumulation was observed (Fig 2E). This strongly suggests for a metabolic connection of these metabolites in *L*. *theobromae* and our results showed that the volume of the culture influences the rate of OPC-4 conversion into JA.

Beside the formation of anabolites of JA, like OPC-4, we as analyzed potential catabolites of JA. In plants, ω-oxidation of its pentenyl-side chain is discussed to be one possible way to inactivate JA-signaling [31–34, 37, 43–45]. In order to further evaluate JA-catabolism and to examine potential parallels between plants and fungal JA-metabolism, we analyzed fungal cultures at different time-points for hydroxy-JA derivatives. Interestingly, we could detect formation of 11/12-hydroxy-JA starting at day 3 (Fig 2F). Based on these findings, we hypothesized that fungal and plant JA-metabolism may involve similar reaction steps.

Several studies reported on the capability of fungal species to produce JA [14, 16, 18, 20, 27, 42, 46]. However, only recently the biotechnological capacity to produce this metabolite has been recognized and attempts to systematically optimize the cultivation conditions were reported [14–16]. In this study, we attempted to model fungal growth and to relate this parameter to JA-production and substrate/glucose consumption. By this analysis, we on the one hand gathered information on parameters of JA-production and fungal growth that may be important for the development and further optimization of fungal cultivation. On the other hand, our results also demonstrate that JA-production is a non-growth related process and thus corroborate that JA is a compound of the secondary metabolism in the fungus.

As mentioned above JA plays a key role in plant development and plant defense and thereby also orchestrates the metabolism of different phytohormones. In order to manipulate those stress- and defense reactions of infected plants on a metabolic level, several fungal species adopted the capacity to produce phytohormones and further plant secondary metabolites (for recent reviews on this topic see [47, 48]). To further test this idea and thereby to gather more information on fungal JA-metabolism, we analyzed whether additional phytohormones and secondary metabolites are produced by *L*. *theobromae* beside JA. Our results demonstrate that *L*. *theobromae* strain 2334 synthesizes several plant related compounds of the secondary metabolism, which were detectable in the culture filtrate. Interestingly, one of those metabolites was identified as chorismate (data not shown). This compound may be regarded as the starting point for tryptophan-biosynthesis and is thus an important constituent of the so-called tryptophan-derived secondary metabolism [30] and also of indole acetic acid (IAA) biosynthesis in plants [49]. In addition, chorismate serves as precursor of salicylic acid, a compound that plays a key-role in the regulation of different physiological processes [50]. Besides minor amounts of chorismate, we found that trace amounts of IAA (0.03–0.07 mg.L^-1^, data not shown) were formed by *L*. *theobromae* strain 2334. Although IAA has been mainly detected in plants, also microorganisms as well as some animals have the capacity to produce IAA (i.e. [51]). In plants, IAA functions as regulator of plant development, whereas in animals antitumor activity has been reported. In addition, antimicrobial activity was observed for some IAA-derivatives [13].

Another metabolite that plays a role in the tryptophan-derived secondary metabolism, which was detected in high amounts in the fungal filtrate, was ICA. This indolic metabolite is formed upon pathogen infection in plants [52] and has also been detected in several microorganisms [53]. It is interesting to note, that two different routes for the biosynthesis of ICA have been proposed for plants and microorganism. While microbial ICA-formation is thought to involve IAA as precursor, plants use a different route that is independent from IAA [52, 53]. Recently, formation of ICA was also observed in *L*. *theobromae* strain ME4-2 that was isolated from floral parts of mistletoe. Since only minor amounts of IAA (0.03–0.07 mg.L^-1^) could be detected in this study, it was proposed that ICA in this *L*. *theobromae* species is formed via an IAA-independent route [54]. This finding may thus suggest an ICA-biosynthetic route that is common for microorganisms as proposed by [53]. In line with the detection of ICA in *L*. *theobromae* strain 2334 is a recent study by Castillo and co-workers who detected indole-3-butyric acid and indole-3-propionic acid—two further indole-derivatives—beside IAA. However, formation of ICA was not reported in that work [21].

About thirty years ago Miersch and co-workers observed formation of (+)-7-iso JA, (+)-9,10-dihydro-7-iso JA, (+)-11,12-didehydro-7-iso JA, and (+)-cucurbic acid as well as a large variety of hydroxylated JA-derivatives including 8-, 11- and 12-hydroxy-JA in the culture filtrate of *L*. *theobromae* strain D 7/2. Later, further JA-derived molecules were identified and termed lasiojasmonates [46, 55] suggesting that a large variety of JA-derived metabolites are formed by *L*. *theobromae*. In order to expand the knowledge on the repertoire of the different jasmonates formed by *L*. *theobromae*-species, we analyzed the main secondary metabolites secreted into the medium. Besides the already reported structures of JA, 9,10-dihydro-JA and 11/12-hydroxy-JA, we identified two further oxidized dihydro-JA-derivatives: hydroxy-9,10-dihydro-JA and dihydroxy-9,10-dihydro-JA. Formation of the analogous 11-hydroxy derivative in *Aspergillus niger* has been reported before [23]. A further oxidized JA-derivative (compound 2a) was detected that gave the same MS/MS-spectrum as 11/12-hydoxy-JA but eluted at an earlier retention-time suggesting an enhanced polarity of this compound. Potential positions for the hydroxyl-group are the C8, C9 and C10 of the JA backbone. Location of the hydroxyl-group at the latter two positions would result in the formation of an enol moiety that might tautomerize to the corresponding keto form via keto-/enol tautomerism. As there is no evidence for a functional keto-group in our analysis and as we anticipate that the resulting keto-tautomere may exhibit a rather increased retention time compared to the hydroxy-isomers, we tentatively assign compound 2a as 8-hydroxy-JA. This idea is supported by the above mentioned study of Miersch and co-workers that showed the formation of 8-hydroxy-JA by *L*. *theobromae* [19]. Unfortunately, no authentic standard was available to test our hypothesis.

An oxygenase that has the capacity to oxidize JA has been identified recently in fungi [56] but remains unidentified in plants. Here, ω-oxidation has only been reported to occur at the respective Ile conjugate—JA-Ile—and is considered as one possibility to inactivate the JA-signaling activity [31, 43, 45]. Formation of JA-Ile and further JA-amino acid conjugates by *L*. *theobromae* strain 2334 (JA-Gly, JA-Ser, JA-Thr) has been reported recently [21]. We did not detect any JA-amino acid conjugate produced by the *L*. *theobromae*-strain used in this study. Castillo and co-workers described the signal of m/z 310.2028 in the supernatant of *L*. *theobromae* strain 2334 as JA-Tyr [21]. We obtained similar mass and fragmentation pattern for compound 7, but the high resolution mass information of our analysis rebut the identity as JA-Tyr. Our analysis indicated that compound 7, one of the main metabolites of the fungal culture filtrate, consisted of Ile-residue that was bound to a C_11_-moiety via an amide-bond and could thus reminiscent to a JA-Ile structure shortened by a CH_2_-moiety.

## Conclusion

In this study, we used the fungus *L*. *theobromae* to produce JA and optimized the cultivation parameters to enhance the final JA-yield. Using those parameters, we obtained from a single 100 mL fungal culture as much JA as that is produced by more than 1000 *Jasminum sambac* blossoms. Furthermore, we establish a mathematic model that describes and correlates fungal growth and JA-production and thus may provide a basis for upscaling processes in industrial fermentation. Our results also demonstrate that beside JA further metabolites are formed that have originally been identified in plants. They suggest that fungal JA-metabolism involves similar reactions as in plants. A deeper understanding of the inherent function of JA and its metabolic pathways in fungi will help to further improve JA production and is an interesting topic for further investigations.

## Supporting Information

S1 Table Raw data table. In this table, the raw data of the different experiments are listed.
(XLSX)
